# Dissolution of Metals (Cu, Fe, Pb, and Zn) from Different Metal-Bearing Species (Sulfides, Oxides, and Sulfates) Using Three Deep Eutectic Solvents Based on Choline Chloride

**DOI:** 10.3390/molecules29020290

**Published:** 2024-01-05

**Authors:** Carlos F. Aragón-Tobar, Diana Endara, Ernesto de la Torre

**Affiliations:** Department of Extractive Metallurgy, Escuela Politécnica Nacional, Ladrón de Guevara E11-253, P.O. Box 17-01-2759, Quito 170525, Ecuador; diana.endara@epn.edu.ec

**Keywords:** deep eutectic solvents, copper leaching, iron leaching, lead leaching, zinc leaching

## Abstract

Nowadays, deep eutectic solvents (DESs) are seen as environmentally friendly alternatives with the potential to replace traditional solvents used in hydrometallurgical processes. Although DESs have been successfully applied in the recovery of metals from secondary sources, there is still innovative potential regarding DESs as green leaching agents applied in the recovery of metals from primary sources like polysulfide ores. This study aimed to evaluate the characteristics of DESs as solvents for some of the main metals present in typical polymetallic concentrates, like Cu, Fe, Pb, and Zn. Thus, three DESs based on choline chloride (ChCl) were prepared: 1:2 ChCl-urea (also known as reline), 1:2 ChCl-ethylene glycol (also known as ethaline), and 1:2 ChCl-glycerol (also known as glyceline). Then, dissolution tests at 30 °C were carried out with these DESs and different metal- (Cu, Fe, Pb, and Zn) bearing compounds (sulfates, oxides, and sulfides). According to the dissolution tests, it was found that the solubility of the studied metals (expressed as g of metal per Kg of DES) was dictated by the bearing species, reaching the dissolution of the metals from sulfates with values as high as two orders of magnitude higher than the metal solubility values for metal oxides and sulfides.

## 1. Introduction

The use of environmentally friendly, safe, non-toxic, and cheap solvents is emerging as one of the major challenges that green chemistry imposes on the metallurgical industry [1]. Nowadays, the recovery of base metals (e.g., Cu, Pb, and Zn) via hydrometallurgical processes uses large amounts of strong acids or bases, including solutions from substances such as H_2_SO_4_, HNO_3_, HCl, NH_3_, FeCl_3_, and CN^−^ [2]. Therefore, the search for greener solvents is currently a priority in the design of new hydrometallurgical processes. Among the alternative solvents with the potential of being incorporated into mineral processing, a group of solvents, named deep eutectic solvents (DESs), promises an environmentally friendly approach to mineral processing, particularly for minerals that are difficult to treat or that consume a lot of energy [3,4].

DESs are generally liquid solvents at room temperature consisting of a mixture of two constituents. The first constituent is named the hydrogen bond acceptor (HBA). HBA substances are ionic components with an anion such as chloride (e.g., choline chloride (ChCl) and other similar quaternary ammonium salts). The second constituent is named the hydrogen bond donor (HBD). Examples of substances used as HBDs include amides (e.g., urea and d trifluoroacetamide (TFA)), alcohols (e.g., ethylene glycol and glycerol), carboxylic acids (e.g., oxalic acid, malonic acid, and lactic acid), or sugars (e.g., fructose, glucose, and sucrose). Since the formed DES is a binary mixture, it maintains the identities of the two components (the HBA and HBD). Thus, the interactions between these two components are through hydrogen bonds and do not form covalent compounds between them [5].

To produce a DES, the proportion of the HBA with respect to the HBD is adjusted considering the formation of a eutectic mixture as the point of departure. A eutectic composition has a lower melting point than any other composition, including the two separate components. A DES is a mixture that has a negative melting point deviation from this ideal eutectic mixture [6]. Simply, any mixture within a certain composition range in the vicinity of the eutectic composition that is in a liquid state at the operating temperature could be used and defined as a DES [6]. A DES is not limited to a mixture in a fixed composition, but a mixture of several compositions that meet the characteristics mentioned above. In fact, by adjusting the composition of its components, the properties of a DES can be tuned for a specific application. This tunability is the basis for the success of DESs in several applications such as reaction solvents [7], the dissolution of major natural biopolymers [8], polyphenol extraction [9], chiral electroanalysis [10], electrochemistry [11], and pharmaceutical applications [12].

DESs are neither novel compounds nor pseudo-pure compounds, but mixtures. Designations found in the literature for these mixtures, such as reline (a 1:2 mixture of ChCl and urea), ethaline (a 1:2 mixture of ChCl and ethylene glycol), glyceline (a 1:2 mixture of ChCl and glycerol), and other similar designations [5], should be taken carefully since they represent the mixtures of known components exclusively in the specified compositions, but they do not suggest new pure components [6].

The use of DESs as leaching agents for the dissolution of metals is presented extensively in the literature. The following studies can be highlighted, such as that presented by Abbott et al. [13], in which metal oxides (CuO, Fe_3_O_4_, and ZnO) were dissolved in a DES formed of chloride of choline and carboxylic acids (malonic acid, oxalic acid, and phenylpropionic acid). Other oxides (TiO_2_, V_2_O_5_, and Cr_2_O_3_) were dissolved in a DES formed of the combination of choline chloride with malonic acid, urea, or ethylene glycol [14]. The solubilization of ZnO from the dust of an electric arc furnace was also possible using a DES composed of ChCl as the HBA and urea or ethylene glycol as the HBD [15].

Additionally, Pateli et al. [16] present electrochemical oxidation as an alternative to the dissolution of metal oxides (MnO_2_, MnO, Fe_2_O_3_, Fe_3_O_4_, Co_3_O_4_, CoO, NiO, CuO, Cu_2_O, ZnO, and PbO) in a DES made up of ethylene glycol and ChCl. Entezari-Zarandi and Larachi [17] studied the dissolution of rare earth carbonates (Y, La, Ce, Nd, and Sm) in a DES formed of ChCl with urea, malonic acid, and citric acid. In that study, the selective dissolution of the rare earth carbonates with higher molecular weights was observed. Another study involving the use of a DES was carried out by Riaño et al. [18] in which a DES based on choline chloride and lactic acid (1:2 molar ratio) was used for the leaching of rare earth elements and other metals from NdFeB magnets.

The dissolution of metals from ores using DESs has been documented in recent years as well. Leaching agents using ethaline (ChCl and ethylene glycol) with iodine (I_2_) as an oxidizing agent at 50 °C have been successfully applied for the dissolution of electrum (gold–silver), chalcopyrite, galena, and native tellurium [4]. In the study by Abbott et al., [19] the influence of ethaline on the electrochemical behavior of pyrite (FeS_2_) at room temperature was presented. Using the same approach as the previous study, Hartley et al. [20] presented the electrodissolution of five iron minerals: pyrite (FeS_2_), marcasite (FeS_2_), pyrrhotite (Fe_1−x_S), arsenopyrite (FeAsS), and loellingite (FeAs_2_). In this same line, the study by Anggara et al. [21] suggested that DESs can help to solubilize and recover high-purity copper directly from three minerals of copper sulfide: covellite (CuS), chalcocite (Cu_2_S), and chalcopyrite (CuFeS_2_).

Currently, the treatment of base metal ores at the industrial level involves a considerable cost of energy and resources, including in hydrometallurgical stages such as leaching, in addition to pyrometallurgical operations such as roasting. Then, leaching using DESs appears to be a technology that is not sufficiently developed, but it can eventually redefine operations in mineral processing, offering great potential for innovation, as well as presenting an ecological solution for mineral treatment. Thus, this study aimed to evaluate the dissolution of metals commonly found in polymetallic sulfides like Cu, Fe, Pb, and Zn using three DESs based on choline chloride (reline (RE), ethaline (ET), and glyceline (GLY)). In this study, different metal-bearing species (sulfides, oxides, and sulfates) were included to evaluate the influence of the chemical speciation on the dissolution of these four metals in the DESs based on choline chloride.

## 2. Results

In this section, the results of the leaching tests (using 0.5 g of powder of the metal-bearing species per 20 g of DES, at 30 °C and for 24 h) are presented as mg of the metal studied (Cu, Fe, Pb, or Zn) per Kg of the DES used (reline (RE), ethaline (ET), or glyceline (GLY)). As the metal-bearing species, sulfides, oxides, and sulfates were used. The solubility of these three species varies according to the way the metal is associated with sulfur (S^2−^) in the case of sulfides, with oxygen (O^2−^) in the case of oxides, and with the sulfate ion (SO_4_)^2−^ in the case of sulfates. Preliminarily, it is known that sulfates are a highly soluble species in water, while oxides and sulfides have limited solubility. In fact, to increase the solubility of these two species (oxides and sulfides), strong acid solutions (for oxides) or even pyrometallurgical methods such as roasting (for sulfides) are applied.

### 2.1. Evaluation of the Quantity of Copper Solubilized in Deep Eutectic Solvents Based on Choline Chloride from Oxide, Sulfide, and Sulfate of This Metal

**Figure 1:**
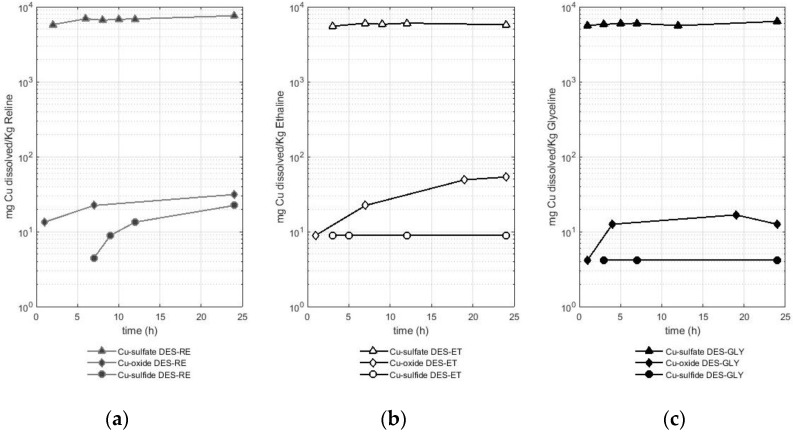
Amount of dissolved copper (mg) from different copper-bearing species (sulfate, oxide, and sulfide) per Kg of deep eutectic solvent in (**a**) RE—reline, (**b**) ET—ethaline, and (**c**) GLY—glyceline.

The first metal studied was copper, and the results of the solubility experiments are presented in Figure 1. Figure 1 compares the different solubilities of copper according to the metal-bearing species in reline (Figure 1a), ethaline (Figure 1b), and glyceline (Figure 1c). A logarithmic scale is used for a better comparison between the results.

As presented in Figure 1, the copper from the sulfate reached values of approximately 5.5 g of copper dissolved per Kg of DES starting from the first hours of the test. This behavior was observed with the three DESs used. Thus, the following average solubility values were reported: 6.7 ± 0.5 g of Cu per Kg of reline, 5.8 ± 0.2 g of Cu per Kg of ethaline, and 5.9 ± 0.3 g of Cu per Kg of glyceline. These solubility values reported for copper from the sulfate are two orders of magnitude higher than the solubility values of copper from the two other metal-bearing species (the oxide and sulfide).

After the sulfate, the oxide was the metal-bearing species that released copper in a greater quantity compared with the sulfide as the metal-bearing species. Reline dissolved between 13 and 31 mg of Cu per Kg of reline when the metal-bearing species was an oxide, while this DES dissolved between 4 and 22 mg of Cu per Kg of reline when the metal-bearing species was a sulfide (chalcopyrite). Ethaline dissolved between 9 and 53 mg of Cu per Kg of ethaline when the metal-bearing species was an oxide, while this DES dissolved 9 mg of Cu per Kg of ethaline when the metal-bearing species was a sulfide (chalcopyrite). Finally, glyceline dissolved between 4 and 17 mg of Cu per Kg of glyceline when the metal-bearing species was an oxide, while this DES dissolved 4 mg of copper per kg of glyceline when the metal-bearing species was a sulfide (chalcopyrite). Therefore, reline is the DES that better solubilized copper from the sulfate or sulfide, while ethaline dissolved more copper when the metal-bearing species was an oxide.

The dissolution of copper in DESs is also reported in the literature, for instance, a value of 6.4 g of copper from an oxide (CuO) per Kg of reline at 50 °C (reported as 0.12 mol Cu dm^−3^ reline) was presented in the study by Abbott et al. [22]. In a later study, Abbott et al. [23] reported a solubility value of 393 mg of copper from an oxide (CuO) per Kg of reline at 60 °C after 48 h of dissolution (reported as 470 ppm). Pateli et al. [16] reported a value of 224 mg of dissolved Cu from an oxide (CuO) per Kg of ethaline (reported as 251 mg Cu L^−1^) after 48 h of leaching at 50 °C. Some variations are observed between the results of Pateli et al. [16] and those reported in this study. As suggested by Pateli et al. [16], the variations in the solubility of the different metals in DESs reported in different studies could be linked to the different methods of chemical analysis, to the differences in the physical properties of the solid powders (i.e., particle size and crystallinity), or even to the water contents of the DESs, where metal oxides are less soluble in aqueous solutions.

Regarding the dissolution of copper from a sulfide (chalcopyrite), the results obtained in this study were compared with those reported by Jenkin et al. [4], in which ethaline was used with iodine (0.1 mol cm^−3^) as an oxidizing agent to dissolve the same mineral. In this study, a dissolution rate of 0.019 µm min^−1^ was estimated. According to our study, the amount of copper reported from the leaching of 507 mg of chalcopyrite in ethaline after 24 h reached a value of 0.18 mg of Cu. As described in Section 4.2, the sulfide samples were pulverized with a referential size of 105 µm. To report values comparable to the data obtained by Jenkin et al. [4], the mineral samples used in the dissolution experiments were assumed to be composed of spheres of 105 µm in diameter. The volume of dissolved chalcopyrite in a DES was calculated considering the referential density of chalcopyrite with a value of 4.19 g cm^−3^ [24]. Thus, it was possible to estimate a value of 1.24 × 10^−5^ µm min^−1^, which is three orders of magnitude smaller than the bibliographic data. This difference may be due to the presence of iodine as an oxidizing agent and opens the possibility of the use of this oxidizing agent to increase the metal recovery in future research.

### 2.2. Evaluation of the Quantity of Iron Solubilized in Deep Eutectic Solvents Based on Choline Chloride from Oxide, Sulfide, and Sulfate of This Metal

**Figure 2:**
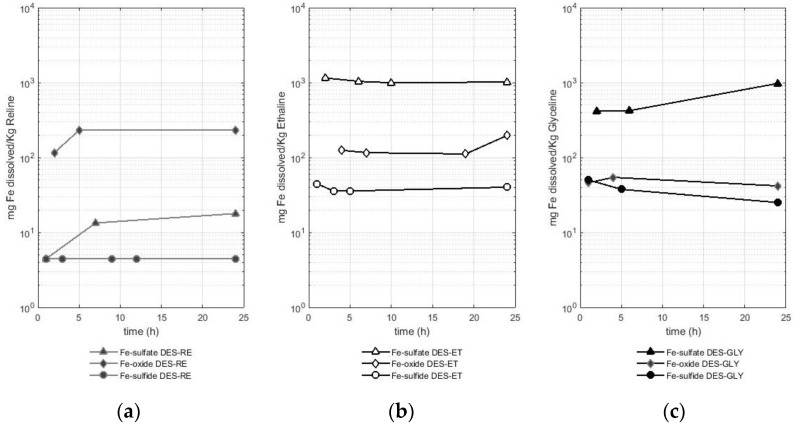
Amount of dissolved iron (mg) from different sources (iron sulfate, iron III oxide, and sulfide of iron pyrite) per Kg of DES in (**a**) RE—reline (**b**), ET—ethaline, and (**c**) GLY—glyceline.

The second metal studied was iron, and the values of solubility for this metal are presented in Figure 2. Figure 2 shows the different solubilities of iron according to the metal-bearing species in reline (Figure 2a), ethaline (Figure 2b), and glyceline (Figure 2c). A logarithmic scale is used for a better comparison between the results.

As presented in Figure 2, the dissolution of iron in reline was lower compared with the dissolution of iron in ethaline and glyceline. In fact, the solubilized amount of iron from the sulfate in reline only reached values between 4.5 and 17 mg of dissolved iron per Kg of DES, whereas values of 0.9 to 1.1 g of solubilized iron per Kg of DES were reported in ethaline, and values between 0.4 and 0.9 g of solubilized iron per Kg of DES were reported in glyceline. Regarding dissolution in ethaline and glyceline, iron from the sulfate dissolved in a greater proportion than iron from the other two metal-bearing species (the oxide and sulfide), reaching values that were one order of magnitude higher.

The other two metal-bearing species (the oxide and sulfide) presented lower solubilities compared with the sulfate. Reline dissolved between 116 and 232 mg of Fe per Kg of reline when the metal-bearing species was an oxide, while this DES only dissolved 4 mg of Fe per Kg of reline when the metal-bearing species was a sulfide (pyrite). Ethaline dissolved between 111 and 196 mg of Fe per Kg of ethaline when the metal-bearing species was an oxide, while this DES dissolved between 35 and 45 mg of Fe per Kg of ethaline when the metal-bearing species was a sulfide (pyrite). Finally, glyceline dissolved between 41 and 54 mg of Fe per Kg of glyceline when the metal-bearing species was an oxide, while this DES dissolved between 25 and 50 mg of Fe per Kg of glyceline when the metal-bearing species was a sulfide (pyrite). Therefore, ethaline is the DES that better solubilized iron from the sulfate and sulfide (pyrite), while reline dissolved more iron when the metal-bearing species was an oxide. The lowest solubility of iron from the sulfide (pyrite) was observed when reline was used as the leaching agent.

The dissolution of iron in DESs is also reported in the literature, for instance, a value of 41 mg of iron from an oxide (Fe_2_O_3_) per Kg of reline at 60 °C after 48 h of dissolution (reported as 49 ppm) was reported in the study presented by Abbott et al. [16]. This value is slightly lower than the ones observed in this study (obtaining values between 116 and 232 mg of Fe per Kg of reline). Regarding iron (from an oxide), dissolution in ethaline (for 48 h and at 50 °C), the study by Pateli et al. [16] reported a value of 0.12 mg of dissolved iron from the oxide (Fe_2_O_3_) per Kg of ethaline (reported as 0.13 mg Fe L^−1^). In this study, the values reported are higher, reaching values between 111 and 196 mg of Fe per Kg of ethaline. In the case of this metal, the reported concentrations of iron are higher compared with those reported in the literature. Higher dissolution of iron in a DES would interfere with the recovery of more valuable metals present in commercial ores, such as copper or zinc.

#### Evaluation of the Quantity of Iron Solubilized in Deep Eutectic Solvents Based on Choline Chloride from Sulfides (Pyrite, Chalcopyrite, and Sphalerite)

**Figure 3:**
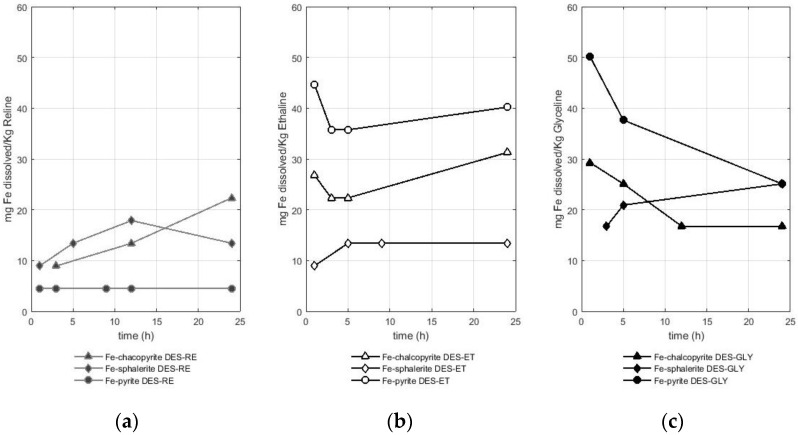
Amount of dissolved iron (mg) from different sulfide minerals (chalcopyrite, sphalerite, and pyrite) per Kg of DES in (**a**) RE—reline (**b**), ET—ethaline, and (**c**) GL—glyceline.

Regarding iron dissolution from sulfides, three metal-bearing species were studied. In addition to pyrite (iron-bearing sulfide), sulfides such as chalcopyrite (copper-bearing sulfide), and sphalerite (zinc-bearing sulfide) also present iron in their compositions. The comparison of the solubility of iron from these sulfides (pyrite, chalcopyrite, and sphalerite) in a DES is presented in Figure 3a for reline, Figure 3b for ethaline, and Figure 3c for glyceline.

Figure 3 shows that the dissolution of iron from the sulfides was between 4 and 50 mg of iron per Kg of the DES used. In fact, the solubility values of iron from the three studied sulfides were in the same order of magnitude. Iron dissolution from pyrite reached values of 4 mg of iron per Kg of reline, 40 mg of iron per Kg of ethaline, and 50 mg of iron per Kg of glyceline. In the case of iron from chalcopyrite, values of 22 mg of iron per Kg of reline, 31 mg of iron per Kg of ethaline, and 29 mg of iron per kg of glyceline were reported. In the case of iron from sphalerite, values of 18 mg of iron per Kg of reline, 13 mg of iron per Kg of ethaline, and 25 mg of iron per Kg of glyceline were reported. Therefore, glyceline is the DES that better dissolved the iron from pyrite and sphalerite, while ethaline dissolved more iron from chalcopyrite.

The DESs used as leaching agents were more efficient in solubilizing iron when this metal came from ores such as chalcopyrite or sphalerite, while the solubilization efficiency of iron from pyrite was lower. In fact, this observation corroborates that pyrite is a more stable sulfide compared with other iron-bearing sulfides such as chalcopyrite and sphalerite [24,25]. Although the amount of sample was the same in the three experiments (~0.5 g), the amount of iron available in pyrite is higher (approximately 0.23 g) compared with that in chalcopyrite (0.15 g) and sphalerite (0.08 g). Therefore, iron dissolves more easily in DESs when other metals such as zinc and copper are found in the mineral structure.

### 2.3. Evaluation of the Quantity of Lead Solubilized in Deep Eutectic Solvents Based on Choline Chloride from Oxide, Sulfide, and Sulfate of This Metal

**Figure 4:**
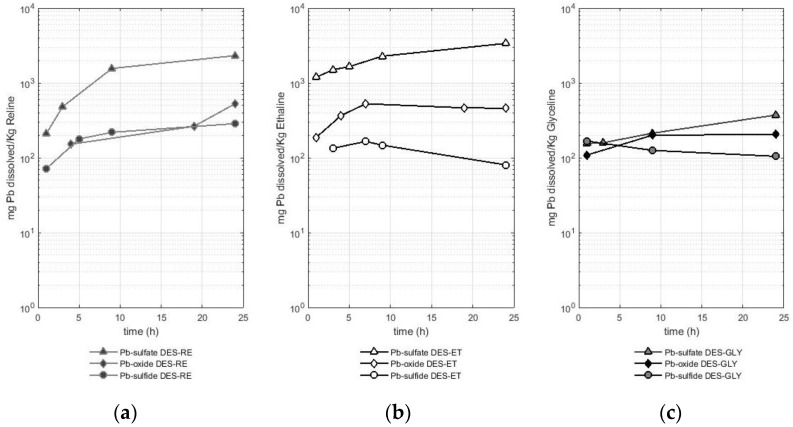
Amount of dissolved lead (mg) from different sources (lead sulfate, lead oxide, and lead sulfide—galena) per Kg of deep eutectic solvent in (**a**) RE—reline (**b**), ET—ethaline, and (**c**) GL—glyceline).

The third metal studied was lead, and the values of solubility for this metal are presented in Figure 4. Figure 4 displays the different solubilities of lead according to the metal-bearing species in reline (Figure 4a), ethaline (Figure 4b), and glyceline (Figure 4c). A logarithmic scale is used for a better comparison between the results.

In a similar way to what was previously observed in copper and iron, the lead solubilized from the sulfate reached values as high as two orders of magnitude higher than the values of the lead solubilized from the sulfide or oxide when ethaline and reline were used as leaching agents, while in the case of leaching with glyceline, similar solubility values were reached for the three species studied. In the case of reline, the solubilized amount of lead from the sulfate reached values between 0.2 and 2.3 g of Pb per Kg of reline. In the case of ethaline, values of 1.2 to 3.4 g of lead solubilized from the sulfate per Kg of ethaline were obtained, whereas the solubility values for glyceline were lower, reaching between 155 and 372 mg of lead solubilized from the sulfate per Kg of glyceline.

The other two lead-bearing species (oxide and sulfide) presented lower solubilities, having a similar tendency toward the dissolution behavior previously observed in copper and iron. Reline dissolved between 151 and 527 mg of lead per Kg of reline when the metal-bearing species was an oxide and between 71 and 286 mg of lead per Kg of reline when the metal-bearing species was a sulfide (galena). Ethaline leached between 187 and 460 mg of Pb per Kg of ethaline when the metal-bearing species was an oxide and between 80 and 165 mg of Pb per Kg of ethaline when the metal-bearing species was a sulfide (galena). Finally, glyceline leached between 109 and 205 mg of Pb per Kg of glyceline when the metal-bearing species was an oxide, while in the case of the sulfide, the amount of lead leached was between 105 and 134 mg of Pb per Kg of glyceline. Therefore, ethaline better solubilized lead from the sulfate, while reline was more efficient in solubilizing lead from the oxide or sulfide. On the other hand, glyceline was the least efficient of the three DESs used to dissolve lead from the three lead-bearing sources (sulfate, oxide, and sulfide).

The dissolution of lead in DESs is also presented in the literature, for instance, a value of 7.66 g of lead from an oxide (PbO_2_) per Kg of reline at 60 °C after 48 h of dissolution (reported as 9157 ppm) was reported by Abbott et al. [23]. This value is 15 times higher than that reported in this study. In the case of the dissolution of lead from oxides in ethaline (for 48 h and at 50 °C), Pateli et al. [16] reported a value of 1492 mg of dissolved lead from an oxide (PbO) per Kg of ethaline (1670 mg Pb L^−1^). This value of lead dissolved in ethaline is twice the amount reported in this study. It is necessary to mention that the experimental conditions reported in the literature involved a higher temperature (50 °C vs. 30 °C in this experiment) and a longer time (48 h compared with 24 h in this experiment). Therefore, the influences of a higher temperature and longer time on the metal recovery are operating parameters that should be investigated in more detail in future studies.

In the case of the dissolution of the lead-bearing sulfide (galena), the results obtained in this study are compared with those reported by Jenkin et al. [4], in which ethaline with iodine (0.1 mol cm^−3^) was used as an oxidant agent to dissolve this mineral. Thus, Jenkin et al. [4] reported a dissolution rate of 0.099 µm min^−1^. In our study, it is known that the greatest amount of lead reported in the solution of galena (514 mg) in ethaline corresponded to 3 h with a value of 2.7 mg of Pb. As mentioned in Section 4.1, the sulfide samples were pulverized, and a referential size of 105 µm was reached. To report values comparable to the data obtained by Jenkin et al. [4], the mineral sample used in the dissolution experiment was assumed to be composed of spheres of 105 µm in diameter. The volume of dissolved galena in the DES was calculated considering the referential density of galena with a value of 7.4 g cm^−3^ [24]. Thus, it was possible to estimate a value of 5.91 × 10^−4^ µm min^−1^, which is two orders of magnitude smaller than the bibliographic data. As previously mentioned in the case of chalcopyrite, the difference with the values found in the literature may be related to the presence of iodine as an oxidizing agent. This finding opens the possibility of the use of this oxidizing agent to increase the metal recovery in sulfides in future research.

### 2.4. Evaluation of the Quantity of Zinc Solubilized in Deep Eutectic Solvents Based on Choline Chloride from Oxide, Sulfide, and Sulfate of This Metal

**Figure 5:**
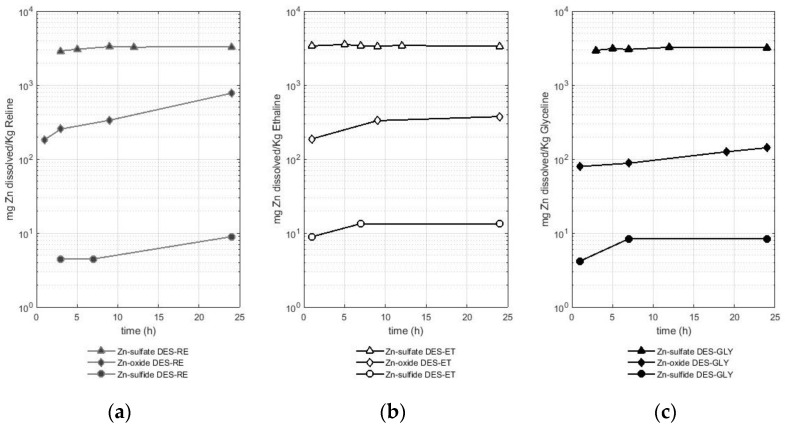
Amount of dissolved zinc (mg) from different zinc-bearing species (sulfate, oxide, and sulfide) per Kg of deep eutectic solvent in (**a**) RE—reline (**b**), ET—ethaline, and (**c**) GLY—glyceline.

The fourth metal studied was zinc, and the results of the solubility experiments are presented in Figure 5. Figure 5 shows the different solubilities of zinc according to the metal-bearing species in reline (Figure 5a), ethaline (Figure 5b), and glyceline (Figure 5c). A logarithmic scale is used for a better comparison between the results.

Figure 5 presents the differences in zinc solubility of up to three orders of magnitude depending on the metal-bearing species. In fact, zinc sulfate is highly soluble in comparison with the oxide and sulfide of this metal, presenting a similar behavior to the dissolution of copper (see Section 2.1). Zinc from the sulfate reached values of approximately 2.9 g of dissolved zinc per Kg of DES in the first hours of the experiment for the three DESs used. Thus, the following average solubility values were reported: 3.2 ± 0.2 g of Zn per Kg of reline, 3.4 ± 0.1 g of Zn per Kg of ethaline, and 3.1 ± 0.2 g of Zn per Kg of glyceline. The solubility values for the zinc sulfate are as high as three orders of magnitude higher if they are compared with the solubility values for the oxide and the sulfide.

Thus, after the sulfate, the oxide leached zinc in a greater quantity compared with the sulfide as the metal-bearing species. The two metal-bearing species (oxide and sulfide) registered lower solubilities, showing a similar trend to that observed in the cases of copper, iron, and lead. Reline dissolved between 183 and 777 mg of Zn per Kg of reline when the metal-bearing species was an oxide and between 4 and 8 mg of Zn per Kg of reline when the metal-bearing species was a sulfide (sphalerite). Ethaline leached between 188 and 375 mg of Zn per Kg of ethaline when the metal-bearing species was an oxide and between 9 and 13 mg of Zn per Kg of ethaline when the metal-bearing species was a sulfide (sphalerite). Finally, glyceline leached between 79 and 142 mg of Zn per Kg of glyceline when the metal-bearing species was an oxide and between 4 and 8 mg of Zn per Kg of glyceline when the metal-bearing species was a sulfide (sphalerite). Therefore, reline managed to solubilize a greater amount of zinc when the metal-bearing species was a sulfate or oxide, while ethaline did so with the zinc from the sulfide. It is also observed that glyceline was the least efficient of the three DESs used.

The dissolution of zinc is also presented in the literature, Abbott et al. [23] reported a solubility value of 7.1 g of zinc from an oxide (ZnO) per Kg of reline at 60 °C after 48 h of dissolution (reported as 8466 ppm). This value is up to 10 times higher than that reported in this study. In the case of zinc dissolution from oxides in ethaline (for 48 h and at 50 °C), Pateli et al. [16] reported a value of 516 mg of dissolved zinc from an oxide (ZnO) per Kg of ethaline (578 mg Zn L^−1^). This value is very close to that found in this study in the same order of magnitude but slightly lower. The differences observed in the case of zinc could be attributed to the lower temperature and shorter time used in our experiments (30 °C and 24 h) compared with the working conditions of 50 °C and 48 h reported in the literature. Therefore, as already mentioned previously for the other metals analyzed, the temperature and the dissolution time are parameters to consider in upcoming investigations.

Therefore, some general characteristics regarding the solubilities of the four studied metals (Cu, Fe, Pb, and Zn) in three different DESs (reline, ethaline, and glyceline) are summarized herein. Comparing the solubilities of the three metal-bearing species (sulfates, oxides, and sulfides), a greater solubility was reached when the metals originated from sulfates instead of the other two metal-bearing species (oxides and sulfides). These differences in solubility reached up to two orders of magnitude for metals such as copper and zinc, and they were less noticeable for iron and lead. Regarding the dissolution of sulfates, reline dissolved copper, iron, and zinc better, while ethaline was more efficient in dissolving lead. In the case of the dissolution of the metals from oxides, reline dissolved the highest amount of iron, lead, and zinc, while ethaline dissolved more copper. Sulfides reported the lowest solubility among the three metal-bearing species studied (sulfates, oxides, and sulfides) for the four metals studied (Cu, Fe, Pb, and Zn), reaching recoveries of less than 5%. However, it is also possible to identify certain patterns in the dissolution of these species. Thus, reline dissolved a higher amount of Cu (from chalcopyrite) and Pb (from galena); glyceline dissolved a higher amount of iron (from pyrite or sphalerite); and ethaline dissolved a higher amount of iron (from chalcopyrite) and zinc (from sphalerite).

## 3. Discussion

### Principles of the Dissolution of Metals from Sulfides, Oxides, and Sulfates in DESs (Reline, Ethaline, and Glyceline)

Common hydrometallurgical operations that use acid and alkaline leaching agents are explained in a more satisfactory way through the mechanisms of the dissolution of metals in these aqueous solvents. Toward this end, the role of the thermodynamics and dissolution kinetics of these metals is undeniable (for example, through properties such as the Gibbs free energy, the relations defining the thermodynamic equilibrium, the Nernst equations, or the Debye–Hückel theory) [26]. However, unlike in aqueous solvents, the principles of the dissolution of these same metals in unconventional solvents such as DESs are still not very well documented in the literature [27,28,29]. This section presents some concepts to better understand the observed behaviors of the dissolved metals in the DESs.

For example, Pourbaix diagrams provide information about the predominant species according to the redox potential (Eh) and the pH of the solution. These diagrams have found applications not only in extractive metallurgy but also in the field of corrosion engineering. The use of these diagrams helps to predict the soluble phases of metals and the conditions of pH and redox potential to promote selectivity between different metal species. Pateli et al. [16] mentioned that the development of Pourbaix diagrams in ionic media such as DESs helps to predict the stable phases of a metal under certain conditions. In fact, the speciation of metals in DESs under different conditions contributes to the design of the beneficiation processes of these metals in DESs. However, not all the thermodynamic data corresponding to the different forms of speciation of metals in these liquids are known. Furthermore, given the wide variety of DESs reported in the literature [5], the task of collecting suitable data for each DES–metal system is challenging nowadays. Furthermore, the value of water activity in DESs is different from the value obtained in aqueous solvents. In fact, the determination of the water activity in each DES imposed an additional difficulty in the task of generating the Pourbaix diagrams for each DES–metal system presented in this study.

The metal-bearing species analyzed in this study (metal sulfates, oxides, and sulfides) have different ionic and covalent characteristics. To dissolve these species in polar media such as DESs, the solvation enthalpy needs to be greater than the lattice energy to form charged species [28]. In fact, this type of interaction has been observed in the recovery of metals (such as Li, Na, Ti, and Al) using molten salts at high temperatures [26].

In the specific case of metal oxides, these compounds are stable due to their strong ionic/covalent characteristics, i.e., high lattice energies, between the oxygen anions and metal cations. The stability of metal oxides is the result of a close-packed crystal structure, which means that the distance between the ions is small, resulting in a higher lattice energy [16].

The phenomena associated with the dissolution of metallic oxides in DESs were explored in the study by Pateli et al. [16]. The study presents the dissolution of oxides in DESs based on choline chloride (HBA) with HBDs such as urea and other HBDs (acids: oxalic, lactic, levulinic, and malonic acid; alcohols: ethylene glycol and glycerol). The study states that the solubility of metal oxides is higher in DESs with acidic HBDs (i.e., oxalic, lactic, and levulinic). In fact, the most acidic DES, such as the eutectic mixture of malonic acid and choline chloride, showed higher solubilities for metal oxides. This behavior observed in DESs with acidic HBDs is linked to the presence of protons (H^+^) that act as oxygen acceptors (O^−2^), breaking the metal–oxide bonds. In the case of DESs with low-proton-activity HBDs, such as those used in this study (ethaline and glyceline), the speciation of metals is primarily governed by the high chloride content (approx. 4.25 M), resulting in chlorine–metal complexes. In the case of reline, there is an additional effect to consider on the metal dissolution related to the formation of metal–amine complexes. The formation of these metal–amine complexes in reline comes from the generation of ammonia due to the decomposition of this DES with heat and humidity. However, at the temperature of the experiments (30 °C), this degradation does not seem to be possible, but it should be considered for leaching experiments at higher temperatures.

Alabdullah [28] proposed a reaction that presents the dissolution of oxides in DESs considering the aspects described in the previous paragraph. The speciation of metals dissolved in DESs was studied using UV–visible spectroscopy coupled with EXAFS (extended X-ray absorption fine structure). In most of the samples analyzed by Alabdullah [28], the dissolved metallic ions appeared as complexes of tetrachloride, indicating that the main factors in the dissolution of these metal oxides are a combination of pH and surface complexation, followed by ligand exchange. Thanks to this characterization, Alabdullah [28] proposed the following dissolution reaction for cupric oxide with the formation of the tetrachloridocuprate (II) ion (CuCl_4_^2−^), which is presented in Equation (1) below:

CuO + 2H^+^ + 4Cl^−^ ↔CuCl_4_^2−^ + H_2_O, (1)

According to Equation (1), the process of dissolution involves proton transfer, so the concentration of H^+^ plays an important role. However, the concentration of H^+^ in a DES is not simply related to pH measurements determined with traditional methods (for example, pH test strips). In fact, the determination of pH in DESs can be difficult to interpret as it implies the activity of protons in non-aqueous solvents [30]. To provide a referential pH value for the DESs used in this study, the approach given by Alabdullah [28] for pH determination was adopted. According to Alabdullah [28], ethaline and glyceline have a neutral pH, while reline has a basic pH. The determination of the pH values of the DESs in the study by Alabdullah [28] goes beyond the simple determination of pH in an aqueous solution. These values were obtained from the absorbance ratios between the protonated and deprotonated forms of an indicator in the respective DESs.

Another factor affecting the activity of the H^+^ that participates in Equation (1) is given by the nature of the HBD that makes up a DES. Alabdullah [28] mentions that the HBD (urea, ethylene glycol, or glycerol) may affect the activity of H^+^ since it affects the activity of chloride ions through the strength of the interaction between the quaternary ammonium from the choline chloride and the hydrogen bond donor (urea, ethylene glycol, or glycerol).

The second parameter to consider in Equation (1) is given by the activity of Cl^−^. The activity value of Cl^−^ can be considered as one in a DES due to their excess in comparison with the other reagents [16]. Therefore, when comparing the H^+^ and the Cl^−^ present in Equation (1), the activity of H^+^ is the main limiting factor for the dissolution of metallic oxides in DESs constituted of poorly coordinated HBDs, as in the case of ethylene glycol (in ethaline) or glycerol (in glyceline).

Regarding the dissolution of other metals in DESs, Alabdullah [28] mentions that establishing similar equations to that presented before (Equation (1)) requires additional effort, since the speciation of other metals is less obvious. In fact, in the dissolution of other metals, the presence of additional ligands was observed. These ligands make equilibrium more difficult to define with respect to the reaction presented in Equation (1). To better illustrate this point, the dissolution of iron salts (nitrates) to form iron oxide in reline was presented as an example. In the study by Hammond et al. [31], the initial speciation of Fe^3+^ in reline was measured using extended X-ray absorption fine structure (EXAFS). It was shown that iron salts formed octahedral [Fe(L)_3_(Cl)_3_] complexes, where L represents various O-containing ligands. Additionally, it was observed that Fe^3+^ induced subtle structural rearrangements in the DES due to the abstraction of chloride into complexes and the distortion of H-bonding around these complexes [31]. Therefore, presenting dissolution reactions like Equation (1) for the other three metals (Fe, Pb, and Zn) is still challenging.

Regarding the dissolution of sulfides in DESs, Carlesi et al. [32] presented some highlights about the dissolution of a concentrate of chalcopyrite in ethaline. They observed that copper and iron from the sulfide dissolved in their original oxidation states, without any change in H^+^ activity, and formed complexes with chloride. Copper was stabilized as a cuprous complex (CuCl_2_^−^). Meanwhile, sulfur was stabilized in ionic form without the in situ formation of elemental sulfur or hydrogen sulfide. These findings suggest that the chemical equilibria of metal chloride complexes limit the dissolution of sulfides in DESs. Moreover, there is a controlling mechanism of this reaction influenced by the interdiffusion of these complexes in DESs. The influence of surface phenomena on sulfide dissolution in DESs is also discussed, presenting the initial chloride adsorption on positive sites of the sulfide surface as part of the mechanism of dissolution. Although the study by Carlesi et al. [32] provides valuable information to complement the findings presented in this study about the dissolution of metals from sulfides, there are still aspects that remain unsolved for future research. For instance, the chemistry of metal complexes is needed to determine the nature of the metal (Cu and Fe) complexes formed and sulfur species solubilized in DESs. In addition, it is necessary to evaluate the importance of chlorine ion adsorption on the positive sites of sulfur particles.

To evaluate if the DESs used in this study can be considered green solvents, recyclability is an aspect that needs to be addressed. This aspect is crucial to present these solvents as alternatives to traditional solvents employed in the recovery of the base metals. Although this topic is outside the main scope of this study, a basic approach is provided. After obtaining a DES with dissolved metals, the following step would be the recovery of these dissolved metals. Some techniques are mentioned in the literature, including precipitation, crystallization, liquid–liquid extraction, or electrochemical deposition [33]. After this stage, the recyclability of these treated solvents can be tested with the regenerated DES in new leaching cycles using the same experimental approach described in this study. It is also suggested that the chemical structure of the pure and regenerated DES can be verified using proton nuclear magnetic resonance, as reported in the literature [34]. However, this additional determination is outside the main scope of the research presented. The study of the recyclability of the DESs obtained in this study opens new insights for future research on metal recovery using DESs.

The findings presented in this study manifest that data related to the dissolution of metals in DESs have not been entirely compiled or developed, even after more than 20 years of research, in the scientific community. However, the authors of this study stand for an optimistic point of view regarding the future of DESs in hydrometallurgy. In fact, the need for a more sustainable approach to hydrometallurgy has promoted the study of alternative solvents like DESs. As pointed out by Binnemans and Jones [35], one of the pillars of circular hydrometallurgy is the use of benign chemicals. In a recent opinion article presented by Binnemans and Jones [29], the mismatch between academic research and the industrial applicability of ionic liquids (ILs) and DESs was highlighted, regarding the insufficient data for engineering purposes and lack of industrial flowsheets. These issues concerning DESs based on choline chloride were briefly discussed in this study, finding some shared views with Binnemans and Jones [29]. In contrast with these two authors who recommend the redirection of the expertise gained on ILs and DESs toward other hydrometallurgical solutions, the authors of this study rely on the future development of the knowledge necessary to overcome the limitations of these solvents, at least those based on choline chloride.

## 4. Materials and Methods

### 4.1. Preparation of Three Deep Eutectic Solvents Based on Choline Chloride (Reline, Ethaline, and Glyceline)

The deep eutectic solvents (DESs) were prepared using choline chloride (analytical grade, 98% purity; Sigma Aldrich, St. Louis, MO, USA) as a hydrogen bond receptor (HBA) in combination with three different hydrogen bond donor (HBD) substances such as urea (analytical grade, 99% purity; Sigma Aldrich), ethylene glycol (analytical grade, 99% purity; Mallinckrod, Staines-upon-Thames, UK), and glycerol (analytical grade, 99.5% purity; Fisher Scientific, Hampton, NH, USA). The first DES prepared was reline (RE), which was composed of a mixture of 1 mol of choline chloride with 2 moles of urea. The mixture formed of these two solid substances was stirred at 80 °C until a transparent liquid was formed. Then, the obtained DES (reline) was cooled to room temperature (15 °C). The same procedure was repeated to obtain the other two eutectic solvents: ethaline (ET) (a mixture of choline chloride and ethylene glycol) and glyceline (GLY) (a mixture of choline chloride and glycerol). These DESs (ET and GLY) were prepared using the same proportion of 1 mole of choline chloride for every 2 moles of ethylene glycol or glycerol, respectively. The preparation protocol for these DESs was developed considering the methodology described in other sources such as [1,5].

### 4.2. Leaching Tests of Different Metal-Bearing Species (Sulfides, Oxides, and Sulfates) Using Deep Eutectic Solvents (Reline, Ethaline, and Glyceline)

In this section, we discuss the dissolution behavior of metals commonly found in commercial ores, like Cu, Fe, Pb, and Zn, studied through leaching tests using the three DESs (RE, ET, and GLY) prepared as described in the previous Section 4.1. As sources of these four metals, three representative bearing species were chosen: sulfides (A_m_S_n_, in which A is the metal studied, and S is sulfur), oxides (A_m_O_n_, in which A is the metal studied, and O is oxygen), and sulfates (A_m_(SO_4_)_n_, in which A is the metal studied, S is sulfur, and O is oxygen).

As bearing sulfides of the studied metals, the following minerals were selected: chalcopyrite (CuFeS_2_) as the copper-bearing sulfide, pyrite (FeS_2_) as the iron-bearing sulfide, galena (PbS) as the lead-bearing sulfide, and sphalerite (Zn,Fe)S as the zinc-bearing sulfide. The sulfide samples were obtained from hand samples of the mineral collection of the Department of Extractive Metallurgy (Escuela Politécnica Nacional). Initially, pieces of approximately 3 g were removed from hand samples in which macroscopic analysis criteria (i.e., brightness, density, and cleavage) revealed the majority presence of the required minerals. The samples were subsequently subjected to a pulverization process, reaching a size of 105 µm. To verify the mineralogical compositions of these samples, X-ray diffraction analysis was performed with a Bruker AXS equipment D8 Advance model (Bruker, Karlsruhe, Germany). From this analysis, it was known that the sample of chalcopyrite was 97% this mineral and 3% pyrite, the sample of pyrite was between 99 and 100% this mineral, the sample of galena was 99% this mineral and 1% plagioclases ((Na,Ca)Al(Si,Al)Si_2_O_8_), and the sample of sphalerite was 98% this mineral and 2% quartz (SiO_2_).

As bearing oxides of the studied metals, the following species were selected: cuprous oxide (CuO, Merck 99% purity, Rahway, NJ, USA) as the copper-bearing oxide; ferric oxide (Fe_2_O_3_, BDH 85% purity) as s iron-bearing oxide, lead dioxide (PbO_2_, Fluka 98% purity) as the lead-bearing oxide, and zinc oxide (ZnO, Merck 99% purity) as the zinc-bearing oxide. Finally, as metal-bearing sulfates, the following species were selected: cupric sulfate pentahydrate (CuSO_4_ 5H_2_O, BDH analytical grade) as the copper-bearing sulfate, ferric sulfate (Fe_2_(SO_4_)_3_ xH_2_O, Panreac 75% purity) as the iron-bearing sulfate, tribasic lead sulfate (PbSO_4_ 3PbO_2_, SULDESA 99.9% purity) as the lead-bearing sulfate, and zinc sulfate heptahydrate (ZnSO_4_ 7H_2_O, Merck 99% purity) as the zinc-bearing sulfate.

Then, the leaching tests were conducted with the samples of the sulfides, oxides, and sulfates of the studied metals (Cu, Fe, Pb, and Zn), using the three DESs (reline—RE, ethaline—ET, and glyceline—GLY) as leaching agents. For each agitated leaching test, 0.5 g of each metal-bearing sulfide, oxide, or sulfate was mixed in 20 g of the corresponding DES at 30 °C and 100 RPM for 24 h. The concentrations of the studied metals in each DES were determined via atomic absorption with a Perkin Elmer AA 300 equipment (Perkin Elmer, Shelton, CT, USA), taking a similar methodological approach as the one presented by Hartley et al. [20]. For this purpose, 100 µL of enriched solution was taken at different times (1, 3, 5, 7, 9, 12, and 24 h). Each sample was first filtered and then diluted to 50 mL using a 2% HNO_3_ solution (for the analysis of Cu, Pb, and Zn) or a 1% HCl solution (for the analysis of Fe).

## 5. Conclusions

Solubility tests (using 0.5 g of powder per 20 g of DES, at 30 °C and for 24 h) were performed with three metal-bearing species (sulfides, oxides, and sulfates) of four metals (Cu, Fe, Pb, and Zn) in three DESs (reline, ethaline, and glyceline). The solubility of the sulfates in the studied DESs reached up to two orders of magnitude higher than the solubility of the oxides and metal sulfides of these metals.

Regarding the solubility of the sulfates in the DESs, reline dissolved higher amounts of copper (6.7 g Cu/Kg reline), iron (17 mg Fe/Kg reline), and zinc (3.2 g Zn/Kg reline), while ethaline dissolved more lead (3.4 g Pb/Kg ethaline).

Regarding dissolution from the metal oxides, reline dissolved the highest amount of iron (232 mg Fe/Kg reline), lead (527 mg Pb/Kg reline), and zinc (777 mg Zn/Kg reline), while ethaline dissolved more copper (53 mg Cu/Kg ethaline).

As for the sulfides, reline dissolved a greater amount of copper (22 mg Cu/Kg reline from chalcopyrite) and lead (286 mg Pb/Kg reline from galena); glyceline dissolved the greatest amount of iron (50 mg Fe/Kg glyceline from pyrite or 25 mg Fe/Kg glyceline from sphalerite); and ethaline dissolved a higher amount of iron (31 mg Fe/Kg ethaline from chalcopyrite) and zinc (13 mg Zn/Kg ethaline from sphalerite).

The differences in the solubility values reported in this study compared with values found in the literature showed the influences of experimental parameters such as temperature, the presence of oxidizing agents (i.e., iodine), the particle size of the powder, and the dissolution time. Studying the influences of these parameters on metal recovery in DESs would complement the insights given in this study.

In a general way, the chemical reaction describing the dissolution of the metal-bearing species studied (Cu, Fe, Pb, and Zn) in the three DESs (1:2 ChCl:urea, 1:2 ChCl-ethylene glycol, and 1:2 ChCl-glycerol) mainly involved the activity of Cl^−^. As products, dissolved species in the form of metallic complexes of chloride adopt different forms according to the metal-bearing species involved.

## Data Availability

Data are contained within the article.
